# Impact of Flexor Tendon Traction Tenolysis on Clinical Outcomes in Open A1 Pulley Release for Trigger Finger

**DOI:** 10.1016/j.jhsg.2024.09.010

**Published:** 2024-11-08

**Authors:** Shalimar Abdullah, Syed Jeffrey Syed Ahmad Kabeer, Lim Chia Hua, Jamari Sapuan, Parminder Singh Gill, Elaine Soh Zi Fan

**Affiliations:** ∗ Department of Orthopaedics and Traumatology, Faculty of Medicine, Universiti Kebangsaan Malaysia, Kuala Lumpur, Malaysia

**Keywords:** A1 pulley, Open release, Stenosing tenovaginitis, Synovium sheath, Traction tenolysis

## Abstract

**Purpose:**

This study aims to compare the postoperative clinical outcomes of open A1 pulley release with and without flexor tendon traction tenolysis. Outcomes assessed include finger range of motion, hand function (Disabilities of the Arm, Shoulder, and Hand [*Quick*DASH] score), complications (eg, digital nerve injury, superficial infection, and residual trigger finger), and surgery duration.

**Methods:**

A prospective study was conducted from January 2018 to June 2019, involving patients with grade II–III trigger finger requiring surgical intervention. Patients were randomized into two groups: group I (open A1 pulley release with flexor tendon traction tenolysis) and group II (open A1 pulley release without flexor tendon traction tenolysis). Postoperative assessments were conducted at 2 weeks, 2 months, and 4 months, documenting finger range of motion, *Quick*DASH scores, complications, and surgery duration.

**Results:**

A total of 50 patients met the selection criteria, with 46 completing the study. The majority were women, with an average age of 56 ± 9.6 years. The patients were predominantly diabetic with the condition affecting the left hand and middle finger and presenting as grade III trigger finger. Baseline characteristics, including age, gender (female/male), ethnicity, occupational status, diabetes status, and trigger finger severity, were comparable between the two groups. Preoperative *Quick*DASH scores were also similar. The mean preoperative finger range of motion at the metacarpophalangeal and proximal interphalangeal joint were lower in group I but were not statistically important. Patients in group I exhibited consistently better postoperative finger range of motion and *Quick*DASH scores compared to group II throughout the follow-up period. The difference was statistically important at the 2-week follow-up. Although group I continued to show better outcomes at 2 and 4 months, the differences were not statistically important. Surgery duration was importantly longer in group I (16.4 ± 5.7 minutes) compared to group II (11.43 ± 3.8 minutes). Two patients in group I experienced wound infections, which resolved with a week-long course of antibiotics.

**Conclusions:**

Open A1 pulley release with flexor tendon traction tenolysis resulted in better early postoperative (2 weeks) finger flexion range of motion and *Quick*DASH scores, albeit with a longer surgery duration.

**Type of study/level of evidence:**

Therapeutic Ib.

Trigger finger, or flexor tenosynovitis of the flexor tendon and sheath, is a prevalent hand disorder commonly encountered in orthopedics clinics. It affects approximately 2% of the general population and 20% of diabetic patients.^1^ Trigger finger arises from a mismatch between the flexor tendon and the stenotic A1 pulley through which it glides.^2^ The clinical presentation of trigger finger is classified according to the Green classification.^3^ Treatment for trigger finger varies based on the disease stage and can be conservative or surgical. Conservative treatments include activity modification, analgesia, hand therapy, splinting, and corticosteroid injections.^2^ Surgical treatment typically involves open or percutaneous A1 pulley release.

Open trigger finger release provides adequate exposure for A1 pulley release, minimizing the risk of neurovascular injuries associated with percutaneous release. Traditionally, this procedure involved only cutting the A1 pulley. However, in adults, the pathology often includes thickening of the synovial sheath, necessitating its release via traction tenolysis. This additional step, suggested by some authors, ensures complete release.^2^, ^3^, ^4^, ^5^ Traction tenolysis involves retracting the flexor tendons out of the wound using a tendon hook and separating the sheath between the tendons (Fig. 1).

**Figure 1:**
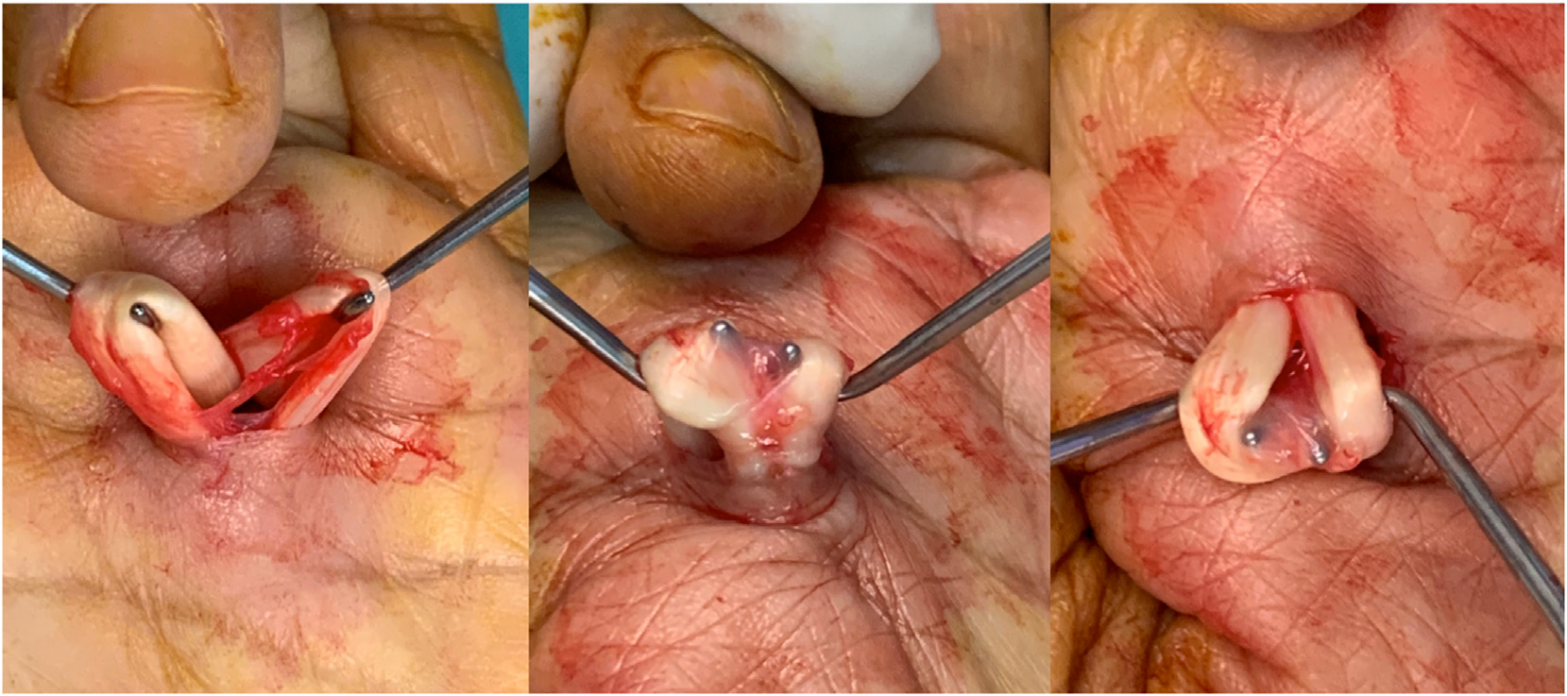
Traction tenolysis releasing the thickened synovial sheath between the flexor pollicis longus and flexor pollicis superficialis tendons.

Restricted flexor tendon motion through a narrowed A1 pulley causes the synovial sheath of both the flexor digitorum superficialis and profundus tendons to adhere to each other (Fig. 1). We hypothesize that the thickening of the tendon sheath between these tendons after open A1 pulley release decreases flexor digitorum tendon excursion, thereby reducing finger active flexion range of motion and postoperative hand functional recovery. We believe that releasing the sheath between the tendons will ensure smooth tendon gliding post-A1 pulley release.

Despite the commonality of open trigger finger release, there is surprisingly scant literature on the traction tenolysis of the flexor tendon. This additional step has been suggested by some authors, yet only one retrospective study by Choudhry et al^4^ has investigated its outcomes, highlighting the need for further research.^2^^,^^3^^,^^5^ Therefore, we aim to conduct a randomized controlled trial comparing open trigger release with and without traction tenolysis to provide more robust evidence on this surgical technique's efficacy.

## Materials and Methods

### Study design and duration

This prospective study was conducted from January 2018 to June 2019. The study included patients over 18 years old with grade II–III trigger finger who required surgery at our center. Exclusion criteria included patients with trigger thumb, recurrent trigger finger, trigger finger involving multiple digits, preexisting hand, finger or rheumatoid hand deformity, history of hemodialysis for renal failure, previous hand surgery, or those opting for percutaneous release.

### Patient selection and consent

All eligible patients received an information sheet and were given ample time to provide informed consent and the study has been approved by our ethics committee (registration ID NMRR-17-3548-39506 [IIR]). Upon consent, patients were assigned an individual research identification number. Demographic data collected included age, gender, ethnicity, hand dominance, occupation, affected hand and finger, duration of symptoms, and grading of trigger finger based on the Green classification.

### Randomization and blinding

Selected patients were randomized into one of two groups using simple randomization:

• Group I: Open A1 pulley release with flexor tendon traction tenolysis
• Group II: Open A1 pulley release without flexor tendon traction tenolysis

Patients were blinded to the type of surgical intervention received. All surgeries were performed by a single surgeon under local anesthesia. The duration of surgery was recorded.

### Surgical technique

All traction tenolysis procedures in patients in group I were performed by a single surgeon (J.S.), involving retracting the flexor tendons out of the wound using two tendon hooks and separating the sheath between the tendons by traction. Some thickened tenosynovial sheaths which cannot be released with traction alone were separated with Steven scissors. To ensure a complete release was performed, each tendon was then independently withdrawn from the wound to be checked. Patients were also instructed to make a fist and a palm at the end of the procedure to ensure complete release.

### Follow-up and outcome measures

Patients were reviewed at 2 weeks, 2 months, and 4 months after surgery. During each follow-up, the following were documented:

• *Quick*DASH score
• Complications
• Active finger flexion range of motion at the metacarpophalangeal joint, proximal interphalangeal joint, and distal interphalangeal joint using a goniometer

### Statistical analysis

Data were analyzed using the Student *t* test to compare continuous variables, and chi-square or Fisher’s exact test for categorical variables. A *P* value of <.05 was considered statistically significant. By conducting this randomized controlled trial, we aim to provide robust evidence on the efficacy of flexor tendon traction tenolysis in patients undergoing open A1 pulley release for trigger finger.

## Results

### Patient demographics and distribution

A total of 46 patients, each with one affected trigger finger, were included in this study. The cohort comprised 34 females and 12 males with a mean age of 56 years (standard deviation 9.6, range 34–80 years). Patients had been symptomatic for an average of 7.2 months (standard deviation 5.6 months, range 1–24 months) before presenting to the clinic. The left hand was more commonly involved (56.5%), and a majority of the patients had diabetes (63%). The middle finger was the most frequently affected digit (52.2%). The majority of patients presented with grade III trigger finger (73.9%), with no cases of grade IV trigger finger observed (Table 1).

**Table 1:** Demographics and Preoperative QuickDASH Score and Finger Flexion

Patient Demographics	Group I (n = 23)	Group II (n = 23)	*P* Value
Age (y)	55.9 ± 11.2	56.2 ± 7.9	.93
Gender			
M	4	8	.179
F	19	15	
Ethnicity			
Malay	12	7	
Chinese	5	7	.325
Indian	6	9	
Occupation			
Working	9	10	.765
Not working	14	13	
Diabetes			
Yes	14	15	.512
No	9	8	
Duration of trigger finger (months)	7.6 ± 6.8	6.7 ± 4.3	.59
Affected trigger hand			
R	11	9	.552
L	12	14	
Grade of trigger finger			
Grade II	5	7	.502
Grade III	18	16	
Grade IV	0	0	
Preoperative *Quick*DASH score	45.3 ± 20.6	34.9 ± 15.9	.06
**Preoperative finger flexion**			
**MCP joint**	32.5 ± 20.8	35.2 ± 21.2	.67
**PIP joint**	63.7+/-25.0	64.7 ± 25.5	.88
**DIP joint**	53.7 ± 21.8	44.6 ± 20.3	.15

DIP, distal interphalangeal; MCP, metacarpophalangeal; PIP, proximal interphalangeal.

### Comparability of groups

Demographic and clinical characteristics were comparable between group I (open A1 pulley release with flexor tendon traction tenolysis) and group II (open A1 pulley release without flexor tendon traction tenolysis) (Table 1). There were no significant differences in age, gender, ethnicity, occupation status, diabetes status, duration of trigger finger, affected hand and finger, or preoperative *Quick*DASH scores. Before surgery, the mean active flexion range of motion at the metacarpophalangeal and proximal interphalangeal joints was lower in group I compared to group II, but the difference was not statistically important.

### Duration of surgery

Patients in group I had a significantly longer surgery time (mean 16.4 minutes, standard deviation 5.7) compared to those in group II (mean 11.4 minutes, standard deviation 3.8) (*P* = .01) (Table 2).

**Table 2:** Postoperative Follow-up Parameters

Follow-up Parameters	Group I (n = 23)	Group II (n = 23)	*P* Value
Finger flexion ROM (2 wk)			
MCP joint	83.6 ± 11.3	65.35 ± 25	.01
PIP joint	88.9 ± 14.7	77.35 ± 15.2	.012
DIP joint	78.96 ± 16	63.9 ± 24.8	.019
Finger flexion ROM (2 mo)			
MCP joint	88.9 ± 3.5	85.1 ± 11.2	.13
PIP joint	98.7 ± 4.6	95.2 ± 8.4	.89
DIP joint	88.7 ± 6.3	83.2 ± 16.1	.12
Finger flexion ROM (4 mo)			
MCP joint	88.9 ± 3.5	85.1 ± 11.2	.13
PIP joint	98.7 ± 4.6	95.2 ± 8.4	.89
DIP joint	88.7 ± 6.3	83.2 ± 16.1	.12
Postoperative *Quick*DASH score			
2 wk	12.8 ± 15	25.2 ± 18.7	.018
2 mo	4.84 ± 12.1	6.91 ± 10.9	.55
4 mo	0.9 ± 4.2	1.6 ± 6.2	.67
Complications			
Wound infection	2	0	
Nerve injury	0	0	-
Contractures	0	0	
Residual trigger finger	0	0	
Duration of surgery (min)	16.4 ± 5.7	11.43 ± 3.8	.01

DIP, distal interphalangeal; MCP, metacarpophalangeal; PIP, proximal interphalangeal.

### Finger active flexion range of motion

**Figure 2:**
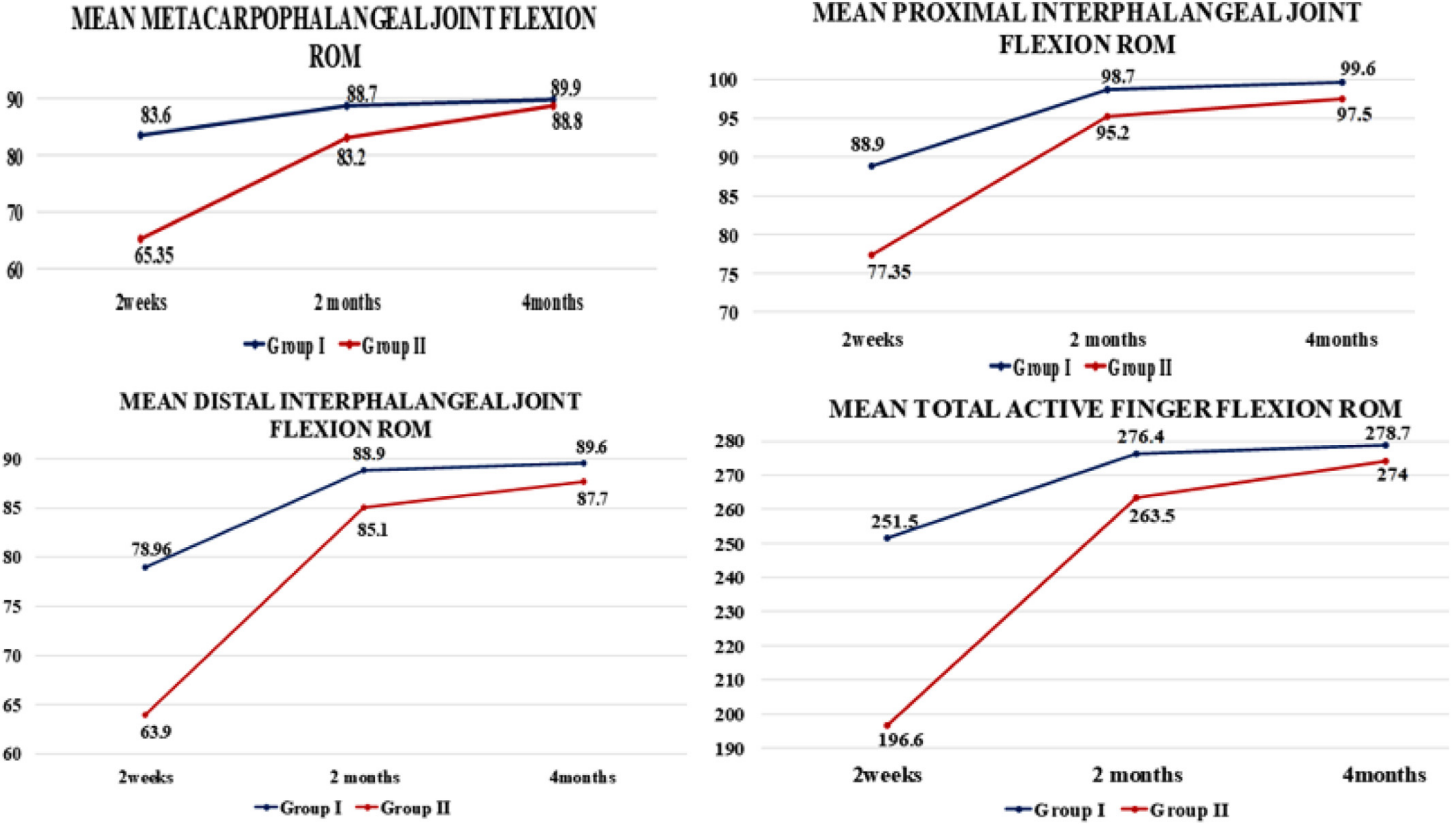
The mean finger flexion range of motion between the two groups at 2 weeks, 2 months, and 4 months.

Before surgery, the mean active flexion range of motion at the metacarpophalangeal and proximal interphalangeal joints was lower in group I compared to group II. After surgery, group I consistently demonstrated higher mean active flexion ranges at the metacarpophalangeal, proximal interphalangeal, and distal interphalangeal joints at all follow-up visits (Fig. 2). The differences in flexion ranges were statistically important at the 2-week follow-up (*P* < .05) (Table 2).

### QuickDASH scores

Before surgery, group I had a higher mean *Quick*DASH score (45.3 ± 20.6) compared to group II (34.9 ± 15.9), although this difference was not statistically significant (*P* = .06). After surgery, group I consistently showed lower *Quick*DASH scores at 2 weeks, 2 months, and 4 months. The difference was statistically significant at the 2-week follow-up (*P* = .018) (Table 2).

### Complications

All patients had resolution of trigger finger symptoms by the end of the 4-month follow-up. Two patients in group I experienced postoperative wound infections, which were successfully treated with a week-long course of antibiotics. No other complications such as nerve injury, finger contractures, or vascular injury were reported in either group throughout the follow-up period.

## Discussion

Trigger finger, or flexor tenosynovitis, is a prevalent hand disorder affecting approximately 2% of the general population and 20% of diabetic patients.^1^ In our study, we observed a higher incidence of trigger finger in females, with the middle finger being the most commonly affected digit, aligning with findings from Lim et al.^6^

Surgical treatment, aimed at releasing the thickened A1 pulley, is indicated for patients with grade III or IV trigger finger or for those with grade II trigger finger who have not responded to conservative treatment.^2^ In this study, we evaluated the additional step of traction tenolysis during open A1 pulley release, a procedure suggested by some authors to improve outcomes by addressing adhesions between the flexor tendons.^2^, ^3^, ^4^, ^5^

A previous retrospective study by Choudhury et al^4^ indicated that patients who underwent traction tenolysis experienced lower total active motion and higher postoperative pain scores. The authors hypothesized that traction tenolysis might trigger an inflammatory response, causing postoperative pain, swelling, and stiffness, ultimately leading to reduced total active motion.^4^ However, the study by Choudhury et al^4^ had several limitations, including its retrospective nature, irregular postoperative follow-up intervals, varying physicians conducting the follow-ups, and significant dropout rates because of inadequate documentation. Additionally, the decision to perform traction tenolysis was at the surgeon's discretion, primarily for patients with residual triggering post-A1 pulley release, and the inclusion of the thumb in the analysis may have skewed the results given its unique anatomy and motion.

Our study addressed these limitations by being prospective, randomized, and blinded concerning the surgical intervention. Regular postoperative follow-ups and consistent range of motion measurements using a goniometer, along with the *Quick*DASH questionnaire for hand function assessment, ensured robust data collection and analysis.

We hypothesized that the inadequate release of adhesions between the flexor tendon synovium sheath during traction tenolysis in the study by Choudhury et al^4^ study might have contributed to their findings. We believe that releasing these adhesions is crucial, as they can limit the excursion of the flexor digitorum profundus tendon because of adherence to the flexor digitorum superficialis, which has a shorter excursion distance.

Our results demonstrated that group I (open A1 pulley release with flexor tendon traction tenolysis) had better mean postoperative finger active flexion range of motion at the metacarpophalangeal, proximal interphalangeal, and distal interphalangeal joints across all follow-ups, with statistically important differences at the 2-week mark. Similarly, group I showed better mean *Quick*DASH scores at 2 weeks, 2 months, and 4 months, with statistically important differences at the 2-week follow-up.

However, the additional step of releasing the flexor tendon synovium sheath adhesions required longer surgical times. Two patients in group I experienced postoperative wound infections, which resolved with a week of antibiotics and were noted to be noncompliant with postoperative dressings.

This study had several limitations. First, the sample size was relatively small, and the study was conducted at a single center, which may limit the generalizability of the findings to the broader Malaysian population or to practices at other centers. Additionally, the surgeon's experience could have influenced the outcomes, introducing potential bias.

The absence of grade IV trigger finger cases in our study restricts the applicability of our results to patients with grade II and III trigger finger only. Although patients were blinded to the type of surgical intervention, the operating surgeon was aware of the procedure being performed, which could introduce performance bias.

Recall and response bias may have occurred during the completion of the *Quick*DASH questionnaire, particularly among patients with high-demand occupations who might report poorer outcomes compared to those with less demanding jobs. Furthermore, preoperative physiotherapy and steroid injection treatments were not documented, which could have influenced the final outcomes.

To enhance the validity and applicability of future research, we recommend studies with larger sample sizes conducted at multiple centers across the country, involving well-trained hand surgeons. Such studies would provide a more comprehensive understanding of the efficacy and outcomes of open A1 pulley release with flexor tendon traction tenolysis.

In conclusion, open A1 pulley release with flexor tendon traction tenolysis demonstrated superior early postoperative outcomes, specifically in finger flexion range of motion and *Quick*DASH scores at the 2-week follow-up. These benefits, however, were not sustained at later follow-ups. The procedure also required a longer surgical duration and was associated with a higher risk of postoperative wound infections. Further research is necessary to evaluate the long-term benefits and risks of this additional step in surgical treatment for trigger finger.

## Conflicts of Interest

No benefits in any form have been received or will be received related directly to this article.

## References

[1] Fitzgibbons P.G., Weiss A.P. (2008). Hand manifestations of diabetes mellitus. J Hand Surg Am.

[2] Ryzewicz M., Wolf J.M. (2006 Jan 1). Trigger digits: principles, management and complications. J Hand Surg Am.

[3] Eastwood D.M., Gupta K.J., Johnson D.P. (1992). Percutaneous release of trigger finger: an office procedure. J Hand Surg Am.

[4] Choudhury M.M., Tay S.C. (2013). Outcome of traction tenolysis in open trigger finger release--a retrospective review. Hand Surg.

[5] Thomas E.T., Ghazi M.R., Thomas E.T., Ghazi M.R., Baratz M.E., Budoff J.E., Slutsky D.J. (2011). Principles of Hand Surgery and Therapy.

[6] Lim M.H., Lim K.K., Rasheed M.Z., Narayanan S., Beng-Hoi Tan A. (2007). Outcome of open trigger digit release. J Hand Surg Eur Vol.

